# Altered microRNA expression in patients with non-obstructive azoospermia

**DOI:** 10.1186/1477-7827-7-13

**Published:** 2009-02-11

**Authors:** Jie Lian, Xiansheng Zhang, Hui Tian, Ning Liang, Yong Wang, Chaozhao Liang, Xin Li, Fei Sun

**Affiliations:** 1 Hefei National Laboratory for Physical Sciences at Microscale and School of Life Sciences, University of Science and Technology of China, Hefei, Anhui 230026, PR China; 2 Departments of Urology, The First Affiliated Hospital of Anhui Medical University, Hefei, Anhui 230022, PR China; 3 Departments of Obstetrics & Gynecology, The First Affiliated Hospital of Anhui Medical University, Hefei, Anhui 230022, PR China

## Abstract

**Background:**

MicroRNAs (miRNAs), a class of small non-coding RNA molecules, are indicated to play essential roles in spermatogenesis. However, little is known about the expression patterns or function of miRNAs in human testes involved in infertility.

**Methods:**

In this study, the miRNA expression profiles of testes of patients with non-obstructive azoospermia (NOA) and normal controls were performed by using microarray technologies.

**Results:**

Altered microRNA expression in NOA patients was found, with 154 differentially down-regulated and 19 up-regulated miRNAs. These findings have been confirmed by real-time reverse transcription-polymerase chain reaction (RT-PCR) assays on select miRNAs, including miR-302a, miR-491-3p, miR-520d-3p and miR-383. Several down-regulated miRNA clusters in patients with NOA were identified, such as the oncogenic potential of the mir-17-92 cluster and mir-371,2,3 cluster.

**Conclusion:**

This is the first report that the expression of miRNAs is altered in testicular tissues of patients with NOA, suggesting a role of miRNAs in regulating spermatogenesis in human males.

## Background

Infertility is a worldwide reproductive health problem which affects 10%–15% of couples. Half of the cases are due to male factors, and about 60–75% of male infertility cases are idiopathic, since the molecular mechanisms underlying the defects remain unknown [1]. A significant proportion of idiopathic male infertility is accompanied by severe oligozoospermia or azoospermia. Spermatogenic cells are characterized with stringently regulated spatiotemporal gene expression and strongly repressed translation in meiotic and haploid male germ cells. For example, impaired chromosome synapsis (marked by synaptonemal complex protein 3 (SCP3) and SCP1) and decreased meiotic recombination (marked by human mutL homologue 1, MLH1, an ortholog of the Escherichia coli Mut L mismatch repair protein) [2-4], were identified in infertile individuals with non-obstructive azoospermia (NOA). Such meiotic errors make these cells susceptible to spermatogenetic arrest and the production of aneuploid gametes. However, the molecular pathways of genetic defects in spermatogenesis are not known. Recently, the mouse maelstrom homolog (MAEL) protein was found in unsynapsed chromosomes of the spermatocyte nucleus and in the chromatoid body (a site where RNA and RNA processing enzymes accumulate) [5]. Furthermore, MAEL interacts with the microRNA (miRNA) pathway-associated proteins mouse vasa homolog (MVH, a germ cell specific RNA helicase), piwi-like homolog 2 (MILI) and piwi-like homolog 1 (MIWI) (Argonaute family members). These observations suggest that miRNAs may be involved in translational repression of meiotic synapsis during spermatogenesis; if so, aberrant miRNA expression would be associated with male infertility.

MicroRNAs are a family of small non-coding RNAs (typically 19–23 nt), which play important roles in regulating post-transcriptional gene silencing through base-pair binding to their target messenger RNAs (mRNAs) [6]. Numerous miRNAs are exclusively or preferentially expressed in the mouse testis [7], suggesting that miRNAs may play important roles in spermatogenesis. A role for miRNAs in translational repression during spermatogenesis has been proposed, which shows that components of the miRNA biogenetic pathway are highly concentrated in chromatoid bodies [8,9]. Transition protein 2 (*Tnp2*), a post-transcriptionally regulated testis-specific gene in postmeiotic germ cells, was found to be regulated by miR-122a [10]. In *Dicer*-deleted testis, spermatogenesis was retarded at an early stage of proliferation and/or early differentiation [11]. Thus, it is reasonable to speculate that miRNAs may be involved in meiotic gene silencing, and that alteration in miRNA expression may be a factor in male infertility. In this study, the expression profiles of miRNAs in the testes of men with NOA and controls were first examined by miRNA microarrays, and the differences in expression levels were verified by quantitative RT-PCR for some of the differentially expressed miRNAs identified. The potential mRNA targets of the miRNAs were predicted using a bioinformatics approach.

## Methods

### Sample collection and RNA extraction

Testicular samples were obtained from the First Affiliated Hospital of Anhui Medical University (Hefei, China). Three patients (ages 22–30 years) with NOA and two patients (ages 60–63 years) undergoing orchiectomy for prostate carcinoma (controls) were recruited for this study. The testicular histology of the NOA patients showed a global early maturation arrest pattern. Two semen analyses indicated azoospermia. An ideal study population as normal controls would consist of volunteers of known fertility, but difficulties in acquiring testicular samples makes this impractical. Instead, samples were analyzed from urology patients who had no history of meiotic defects or infertility and histological examination showed normal spermatogenesis. In addition, none of the controls were exposed to adjuvant hormonal therapy prior to orchiectomy. Informed consent was obtained from all patients, and this study received ethical approval from the institutional review boards of the University of Science and Technology of China and the Anhui Medical University.

Immediately after retrieval, testicular tissues were snap-frozen in liquid nitrogen and stored at -80°C until processed. Total RNA was isolated using Trizol reagent (Invitrogen, USA), following the manufacturer's protocol. The quality of extracted RNA was measured using UV absorbance and denaturing agarose gel electrophoresis.

### MicroRNA microarray analysis

MicroRNA expression profiles of testicular tissues from NOA patients and controls were generated by applying the miRCURY Locked Nucleic Acid (LNA) microarray platform (Exiqon, Denmark). All procedures were carried out according to manufacturer's protocol. Briefly, 1 μg total RNA was dual-labelled with dyes spectrally equivalent to the Cy3™ and Cy5™ fluorophores, using a miRCURY™ Array Power Labelling kit (Exiqon, Denmark). Labelled miRNAs were used for hybridization on a miRCURY™ LNA microRNA Array containing T_m_-normalized capture probes for 582 human miRNAs. Microarrays with labelled samples were hybridized at 56°C for overnight using a heat-shrunk hybridization bag (Phalanx Hybridization Assembly, Phalanx Biotech, Taiwan, China) and washed using miRCURY™ Array Wash buffer kit (Exiqon, Denmark).

Slides were scanned using a Genepix 4000B laser scanner (Axon Instruments, USA) and microarray images were analyzed using Genepix Pro 6.0 software (Axon Instruments, USA). The patients with NOA group and the patients undergoing orchiectomy for prostate carcinoma group, were pooled to represent the study and the control group. Differentially expressed miRNAs were defined as genes whose expression in the study group is consistently altered two-fold (either greater or less) compared to the control group. The 2-fold cut-off is a default for many microarray experiments because it can reflect the variability in the population of samples. Hierarchical clustering for differentially expressed miRNAs was generated by using standard correlation as a measure of similarity.

### Bioinformatics analysis

Genomic coordinates were determined for each miRNA, and mapped to a specific chromosomal region in the human genomes using miRBase: Sequence Release 10.1 [12,13] combined with the UCSC Genome Browser [14]. Patterns of miRNA gene clustering and chromosomal distribution of differentially expressed miRNAs in the human genome were also determined.

To better understand the function of miRNAs, putative mRNA targets of differentially expressed miRNAs were predicted by four algorithms: miRBase Targets [15], TargetScan [16], DIANA-microT [17] and PicTar [18]. The intersections of the four algorithms were obtained from miRGen [19].

### Real-time quantitative PCR

Real-time quantitative PCR was performed to confirm the array results. Reverse transcriptase reactions contained 700 ng of purified total RNA, 20 nM stem-loop RT primer (Table 1), 1 × RT buffer (Epicentre, USA), 0.125 mM each of dATP, dGTP, dCTP and dTTP (HyTest Ltd., Finland), 1 U/μl MultiScribe reverse transcriptase (Epicentre) and 0.6 U/μl RNase Inhibitor (Epicentre, USA). Using the Gene Amp PCR System 9700 (Applied Biosystems, USA), 20 μl reactions were performed with the following thermal cycling parameters: 30 min at 16°C, 42 min at 42°C, 5 min at 85°C and then held at 4°C. All reverse transcriptase reactions, including no-template controls and RT minus controls, were run in duplicate. Reactions for qRT-PCR were conducted in triplicate, using a Rotor-Gene 3000 Realtime PCR instrument (Corbett Research, Australia). Each reaction mixture contained 1 × PCR buffer (Promega, USA), 1.5 mM MgCl_2 _(Promega, USA), 0.25 mM each of dATP, dGTP, dCTP and dTTP (HyTest Ltd., Finland), 1 U DNA polymerase (Promega, USA), 0.4 μM of each primer (Table 1), 0.25 × SYBR Green I (Invitrogen, USA), 1 μl cDNA, and deionized water to a total volume of 25 μl. Reactions were run with the following thermal cycling parameters: 95°C for 5 minutes followed by 40 cycles of 95°C for 10 seconds (denaturation) and 60°C for 60 seconds. The threshold cycle (Ct) is defined as the fractional cycle number at which the fluorescence passes the fixed threshold, and each sample was normalized on the basis of its endogenous U6 RNA content. Data analysis was performed by using Rotor-gene v6.0 software (Corbett Research, Australia).

**Table 1:** Oligonucleotides used in this study.

Primer set name	Reverse transcriptase reaction primer (5' to 3')	Real-time quantitative PCR primer (5' to 3')
U6	CGCTTCACGAATTTGCGTGTCAT	Forward: GCTTCGGCAGCACATATACTAAAAT Reverse: CGCTTCACGAATTTGCGTGTCAT
hsa-miR-491-3p	GTCGTATCCAGTGCGTGTCGTGGAGTCGGCAATTGCACTGGATACGACGTAGAA	Forward: TGCTTATGCAAGATTCCC Reverse: TGCGTGTCGTGGAGTC
hsa-miR-302a	GTCGTATCCAGTGCGTGTCGTGGAGTCGGCAATTGCACTGGATACGACTCACCAA	Forward: GGGGTAAGTGCTTCCATGTT Reverse: CAGTGCGTGTCGTGGAGT
hsa-miR-520d-3p	GTCGTATCCAGTGCGTGTCGTGGAGTCGGCAATTGCACTGGATACGACACCCAC	Forward: GGGGAAAGTGCTTCTCTTTG Reverse: GTGCGTGTCGTGGAGTCG
hsa-miR-383	GTCGTATCCAGTGCGTGTCGTGGAGTCGGCAATTGCACTGGATACGACAGCCAC	Forward: GGGAGATCAGAAGGTGATT Reverse: TGCGTGTCGTGGAGTC3

### *In situ *hybridization

To investigate the cell-specific distribution of miRNA in normal and NOA tissues, *in situ *hybridizations were performed using 5'end digoxigenin (DIG)-labelled LNA modified DNA oligonucleotides (LNAs) complementary to the mature miRNA supplied by Exiqon A/S (Denmark). In this study, the expression of miR-383 was examined in testicular tissues. LNAs had the following sequences: LNA-miR-383, 5'-agccacaatcaccttctgatct-3'. Furthermore, LNA-scrambled, 5'-gtgtaacacgtctatacgccca-3' serves as negative control.

*In situ *hybridization in human testis tissue was performed essentially as described by Obernosterer et al.(2007) [20]. After fixation with 4% paraformaldehyde for 15 min, 10 μm testis sections were immersed and stirred gently in 0.1 M ethanolamine and 2.5% acetic anhydride for 10 min to block endogenous alkaline phosphatase activity. The slides were treated with 5 μg/ml proteinase K for 3 min, followed by extensive washing with PBS. Pre-hybridizations were performed for 6 hr in hybridization oven at a temperature of 52°C with 700 μl pre-hybridization buffer (50% formamide, 5 × SSC, 5 × Denhardt's, 200 μg/ml yeast RNA, 500 μg/ml salmon sperm DNA, 2% Roche blocking reagents and DEPC-treated water). 1 pmol LNA probe was added to 150 μl denaturizing hybridization buffer (50% formamide, 5 × SSC, 5 × Denhardt's, 200 μg/ml yeast RNA, 500 μg/ml salmon sperm DNA, 2% Roche blocking reagents, 0.25% CHAPS, 0.1% tween and DEPC-treated water). After denatured by heating up to 80°C for 5 min and then quickly placed on ice, the probe mixture was pipetted onto the tissues. Glass coverslips were applied, and the slides were hybridized overnight at the pre-hybridization temperature. Sections were soaked in pre-warmed 60°C 5 × SSC to remove the coverslips, and incubated in 0.2 × SSC at 60°C for 1 h. Slides were then incubated in B1 solution (0.1 M Tris pH 7.5/0.15 M NaCl) at room temperature for 10 min. After pretreated in 20% sheep serum (Santa Cruz, USA) diluted with B1 solution at room temperature for 1 hr, the sections were incubated overnight at 4°C in 10% sheep serum containing anti-Digoxigenin-AP FAB fragments (Roche, USA; 1:250). Sections were then washed three times for 5 min in B1 solution at room temperature and equilibrated for 10 min in B3 solution (0.1 M Tris pH 9.5/0.1 M NaCl/50 mM MgCl_2_). Staining with NBT/BCIP (Roche, USA) was done overnight at room temperature. When each probe had yielded a strong signal, or until the negative control began to show background label, reactions were stopped by washing the slides for 3 × 10 min in 1 × PBS. Finally, the slides were mounted with the aqueous mounting medium sealed with nail polish. Signals were visualized under a standard light microscopy.

## Results

### Differential expression of miRNAs between normal and NOA testes

**Figure 1:**
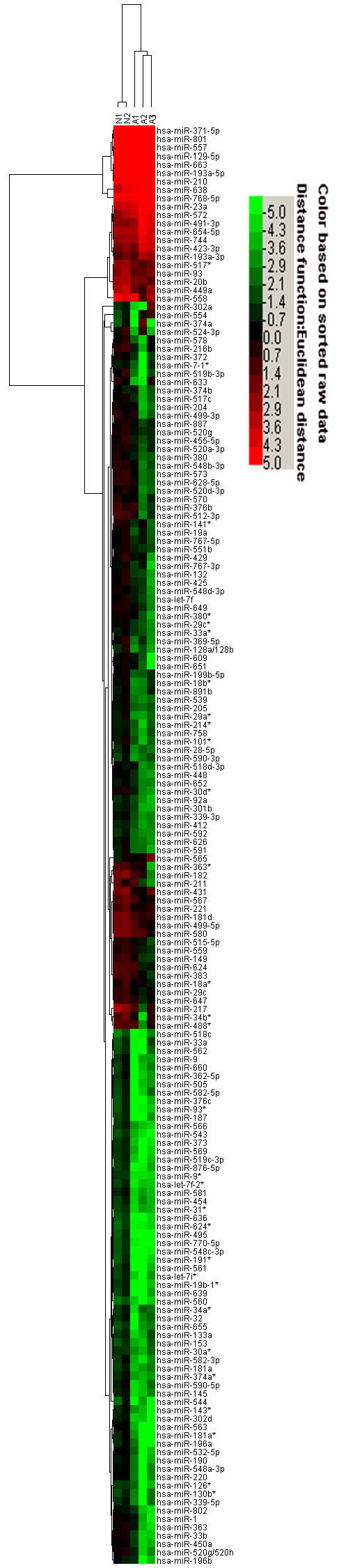
**Hierarchical clustering of miRNA in testicular tissue samples**. Testicular tissue samples were clustered according to the expression profile of 173 two-fold differentially expressed miRNAs between 3 NOA patients and 2 controls. All samples were properly assigned to the correct class. The key color bar indicates that miRNA expression levels increased from green to red color compared to controls (dark color indicates the expression level is close to that of controls). N: controls; A: NOA patients.

By employing a highly sensitive, high-throughput and specific miRCURY™ LNA microarray platform, miRNA expression profiles for testicular tissues were determined from 3 patients with NOA and 2 controls. Among all human miRNAs spotted on the chip, genes were considered to have significant differential expression if they were up- or down-regulated at least two-fold. A total of 173 miRNAs were found to be differentially expressed in NOA patients compared to controls, with 19 up-regulated miRNAs (See additional file 1: Table S1 for Differentially up-regulated miRNAs in NOA patients.) and 154 differentially down-regulated (See additional file 2: Table S2 for Differentially down-regulated miRNAs in NOA patients.). Based on these differentially expressed miRNAs, a tree with clear distinction between control males and NOA patients was generated by cluster analysis (Fig. 1). In addition, at least 7.8% (12) of the 154 differentially down-regulated miRNAs appear to be the testicular miRNAs (Table 2), contrasted with the expression profiles of the mouse testicular miRNAs reported previously [7].

**Table 2:** Summary of down-regulated miRNAs that appear to be the testicular miRNAs^†^

human miRNA	Mean fold-change^††^	Sequence (5'-3')	mouse miRNA	Sequence (5'-3')
hsa-let-7f	-0.33	tgaggtagtagattgtatagtt	let-7f	TGAGGTAGTAGATTGTATAGT
hsa-let-7f-2*	-0.11	ctatacagtctactgtctttcc	*let-7f-1-3p	CTATACAATCTATTGCCTTCCC
hsa-let-7i*	-0.13	ctgcgcaagctactgccttgct	*let-7i-3p	CTGCGCAAGCTACTGCCTTGCT
has-miR-181a	-0.21	aacattcaacgctgtcggtgagt	mir-181c	AACATTCAACCTGTCGGTGAGT
hsa-miR-19a	-0.36	tgtgcaaatctatgcaaaactga	mir-19b	TGTGCAAATCCATGCAAAACTGA
hsa-miR-20b	-0.44	caaagtgctcatagtgcaggtag	*mir-20b	CAAAGTGCTCATAGTGCAGGTA
hsa-miR-29c	-0.45	tagcaccatttgaaatcggtta	mir-29a(+1)	TAGCACCATCTGAAATCGGTTA
hsa-miR-30a*	-0.31	ctttcagtcggatgtttgcagc	mir-30a-3p	CTTTCAGTCGGATGTTTGCAGC
hsa-miR-30d*	-0.23	ctttcagtcagatgtttgctgc	mir-30a-3p	CTTTCAGTCGGATGTTTGCAGC
hsa-miR-34b*	-0.31	taggcagtgtcattagctgattg	mir-34b	TAGGCAGTGTAATTAGCTGATTG
hsa-miR-449a	-0.36	tggcagtgtattgttagctggt	mir-449	TGGCAGTGTATTGTTAGCTGGTTG
hsa-miR-652	-0.35	aatggcgccactagggttgtg	mir-652	AATGGCGCCACTAGGGTTGTGC
hsa-miR-92a	-0.30	tattgcacttgtcccggcctgt	hsa-miR-92	TATTGCACTTGTCCCGGCCTG

^†^Based on expression profiles of the mouse testicular miRNAs from Ro et al. (2007) [7]

^††^Infertile vs. control.

**Figure 2:**
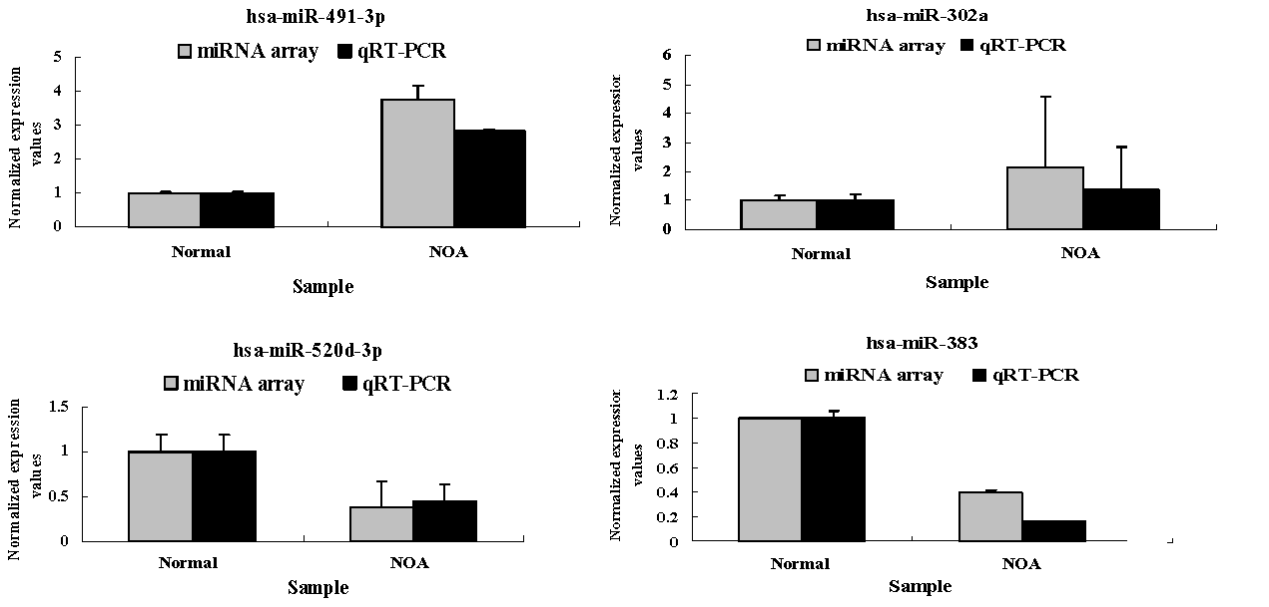
**Confirmation of microarray data by quantitative real-time PCR**. Quantitative real-time PCR analysis confirmed microarray data: miR-302a and miR-491-3p was up-regulated and miR-520d-3p and miR-383 was downregulated in NOA patients. Error bars indicate the SEM.

**Figure 3:**
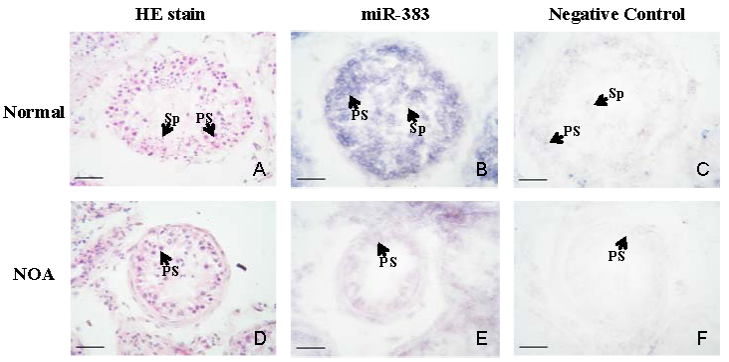
***In situ *hybridization of miRNA in human testes**. *In situ *hybridization analyses using 5' DIG-conjugated, LNA-modified DNA probe complementary to miR-383 was performed on 10-μm frozen sections of the testes of NOA patient and normal control. **A, D: **HE stain of normal (A) and NOA testes (E); **B, E**: In the testis of normal control (B), miR-383 was highly expressed in primary spermatocyte (PS) and lowly expressed in spermatid (Sp); whereas the expression of miR-383 in NOA patient (E) is decreased compared with control. **C, F: **The negative control of normal (C) and NOA testes (F), where the tissues were treated without using DIG-labelled probe. Scale bar = 50 μm.

To confirm the results obtained by microarray analysis, quantitative real-time RT-PCR analysis of normal and NOA testicular samples was performed for miR-302a, miR-491-3p, miR-520d-3p and miR-383. Real-time PCR confirmed microarray analysis results: expression of miR-302a and miR-491-3p was up-regulated (Fig. 2) and miR-520d-3p and miR-383 was down-regulated (Fig. 2) in patients with NOA. Furthermore, *in situ *hybridization data were also verified microarray analysis results: miR-383 expression (major in primary spermatocyte) levels were found to be down-regulated in patients with NOA (Fig. 3).

### Genomic location of differentially expressed miRNAs

**Figure 4:**
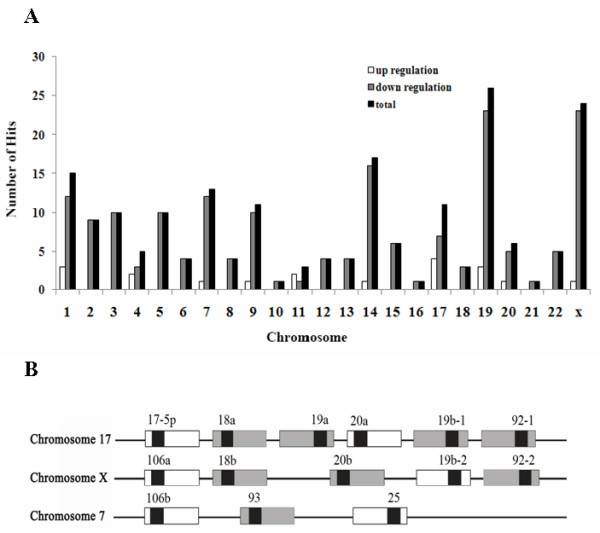
**Genomic locations of differentially-expressed miRNAs associated with spermatogenesis arrest**. **A**. Chromosomal distribution of differentially-expressed miRNA genes identified in patients with NOA. 19 up-regulated (hollow bar) and 154 down-regulated miRNAs (solid bar) were located and numbered on each of the chromosomes. **B**. Chromosomal locations of human *mir-17-92 *family identified in patients with NOA. The *mir-17-92 *family consists of three paralogous clusters: the *mir-17 *cluster (located at chromosome13q31.3), the *mir-106a *cluster (located at chromosome Xq26.2) and the *mir-106b *cluster (located at chromosome 7q22.1). Names of the miRNA precursors are written above the boxes. The position of the mature miRNA is indicated by a dark box. The expression levels of 8 members of this family were differentially down-regulated in testicular tissues for NOA patient (gray), while other members show no significant difference in expression levels between normal and infertile men (white).

Chromosomal locations of miRNA genes have frequently provided important insight into the roles of miRNAs in specific diseases. By mapping differentially expressed miRNA genes onto the chromosomes, genes encoding down-regulated miRNAs were found on all chromosomes but the Y chromosome (Fig. 4A; also see additional file 2: Table S2 for Differentially down-regulated miRNAs in NOA patients.). Of all the chromosomes, chromosomes 14, 19 and X appear to contain the most down-regulated miRNA genes (a total of 62 miRNAs; 35.6%). Chromosomes 10, 16 and 21 contained only one down-regulated miRNA gene. The most up-regulated miRNA genes were located on chromosome 17. Notably, miR-663 was located on chromosome 20 ph (the heterochromatic region on the p arm), in which the genes are usually transcriptionally silent [21].

Clusters of miRNAs within the human genome were also identified. Expressions of 12 of 13 members of a miRNA cluster located at human chromosome 14q32.31 were down-regulated in infertile men, with only miR-654-5p up-regulated (See additional file 3: Table S3 for Several differentially expressed miRNA clusters in NOA patients.). Two miRNA clusters located at chromosome 19q13.42 were identified. One is the *mir-371,2,3 *cluster, consisting of differentially down-regulated miR-372, miR-373 and up-regulated miR-371-5p in patients with NOA; the other cluster, of 13 differentially down-regulated miRNAs (See additional file 3: Table S3 for Several differentially expressed miRNA clusters in NOA patients.), was reported as a new cluster of non-conserved miRNAs [22]. Of particular note is that several members of the *mir-17-92 *family occur relatively infrequently in NOA patients. These miRNAs are encoded by three paralogous clusters located at chromosome 13q31.3 (the *mir-17 *cluster, containing miR-18a*, miR-19a, miR-19b-1* and miR-92-1), chromosome Xq26.2 (the *mir-106a *cluster, containing miR-18b*, miR-20b and miR-92-2) and chromosome 7q22.1 (the *mir-106b *cluster, containing miR-93 and miR-93*) (Fig. 4B) [23].

### Putative miRNA target gene prediction

To determine if miRNA may modulate mRNA levels in infertile testis, four programs (see Materials and Methods) were used to predict targets for differentially expressed miRNAs. Only the targets that were found from at least two of these algorithms were considered, in order to increase the stringency of the analysis. For example, the potential targets for several down-regulated miRNAs, including tissue inhibitor of metalloproteinase 3 (TIMP3), SRY (sex determining region Y)-box 9 (SOX9) and Growth arrest and DNA-damage-inducible, gamma (GADD45G), were shown to be increased in infertile testis (Table 3) [24].

**Table 3:** Potential miRNA targets^† ^that are differentially expressed in infertile testis^††^

miRNA			Potential miRNA targets	
miRNA name	Fold change^†††^	GenBank	Gene name	Fold change^†††^

hsa-miR-1	-0.27	U14394	Tissue inhibitor of metalloproteinase 3 (TIMP3)	+1.77
hsa-miR-181a	-0.21	U14394		
hsa-miR-221	-0.5	U14394		
hsa-miR-9*	-0.1	U14394		
hsa-miR-145	-0.13	Z46629	SRY (sex determining region Y)-box 9 (SOX9)	+1.41
hsa-miR-383	-0.39	AF078078	Growth arrest and DNA-damage-inducible, gamma (GADD45G)	+1.57

^†^Based on the intersections of at least two of four widely used target prediction programs.

^††^Based on RNA expression profile dataset from Rockett et al. (2004) [24].

^†††^Infertile vs. control.

## Discussion

The meiotic and haploid phases of spermatogenesis are characterized by high transcriptional activity but suppressed translational activity. Post-transcriptional control of gene expression in these phases can be mediated by sequences in the 5' and 3'-untranslated regions (UTRs) of mRNAs where they may be regulated by miRNAs, indicating that miRNAs might play important roles in spermatogenesis [25]. In the current study, using the microarray assay, altered expression of miRNAs in the testes of patients with NOA was identified for the first time, suggesting that aberrant miRNA expression may contribute to spermatogenesis arrest in human males.

Many clusters of miRNAs have been identified within the human genome [22,26]. One of the largest miRNA clusters identified to date is hsa-miR-127, a cluster comprised of greater than 50 members residing on an imprinted region of human chromosome 14q32 [22]. Another cluster on chromosome 19 located at positions 58861745–58961404 (HG17), is the largest non-conserved miRNA cluster ever reported and is comprised of 54 miRNAs [22]. Moreover, almost all the known miRNAs encoded by genes on the X-chromosome are expressed in the testis [7,22]. In the present study, preferential down-regulation of miRNAs in chromosomes 14, 19 and X was found in NOA patient testicular tissues. These findings suggest that expression of miRNAs in regions of 14q32.31, 19q13.42 and the X chromosome could be important in spermatogenesis.

Among the differentially expressed miRNAs, it is interesting to note that several members of the *mir-17-92 *cluster and *mir-371,2,3 *cluster are downregulated in NOA testicular tissue. These miRNAs are potential novel oncogenes participating in the development of human testicular germ cell tumors [27,28]. The *mir-17-92 *cluster is highly expressed in carcinoma *in situ *(CIS) testis and inhibits apoptosis by translationally down-regulating E2F transcription factor 1(*E2F1*) in CIS cells [28]. The expression of miR-372 and miR-373 permits proliferation and tumorigenesis of primary human cells by harboring both oncogenic RAS and active wild-type p53 [27]. Therefore, *mir-17-92 *cluster and miR-372/miR-373 may down-regulate apoptosis genes and potentially have a role in inhibiting apoptosis. In this study, low expression of these miRNAs was found in NOA patients, which may contribute to increased apoptosis in the testes of patients with NOA [29].

The prediction of putative mRNA targets for differentially expressed miRNAs will help to characterize the miRNAs involved in biological processes. For example, three possible target genes for down-regulated miRNAs, *TIMP3, SOX9 *and *GADD45G*, were significantly increased in infertile testis [24]. TIMP-3, a potential target of four down-regulated miRNAs (miR-1, miR-181a, miR-221 and miR-9*), is thought to play an active role in testicular development and differentiation [30]. The transcription factor SOX9, a putative target of miR-145, is required for Sertoli cell maturation and normal spermatogenesis [31]. As a potential target of miR-383, GADD45G can induce apoptosis and inhibit cell growth in response to stress shock [32]. Abnormal expressions of these proteins may have a significant impact on male infertility.

During meiotic prophase I of spermatogenesis, synapsis of homologous chromosomes and recombination are essential for the formation of haploid spermatozoa [33,34]. Impaired chromosome synapsis and decreased meiotic recombination can lead to meiotic arrest in spermatogenesis [2,4,35]. In this study, the four differentially expressed miRNAs were chosen to be confirmed in NOA patients by quantitative RT-PCR due to their potential role in regulating meiotic recombination- and synapsis-related genes. For example, one of the predicted target genes of miR-302a and miR-383 is *MLH1*, while miR-491-3p and miR-520d-3p is *SCP1 *and *SCP3*, respectively. The miR-383 was chosen for further *in situ *hybridization analysis because the relative abundance of this miRNA was found be in meiotic prophase [36]. Whether the altered patterns of miRNA expression contribute to defects in chromosome synapsis and recombination in NOA patients awaits further study.

In summary, miRNA expression in testes was profiled and results were compared between patients with NOA and control men. Several miRNA clusters were also examined. However, the cellular origins of testicular miRNAs remain unknown. Azoospermic men with testicular failure have different problems ranging from Sertoli cell-only pattern to maturation arrest, or hypospermatogenesis. Therefore, analysis of the cellular origins and the target genes of these differentially expressed miRNAs in even larger populations of infertile men is warranted in order to shed light on their regulatory roles in spermatogenesis and male infertility.

## Competing interests

The authors declare that they have no competing interests.

## Authors' contributions

JL and XZ carried out the microarray analysis, participated in the design of the study and drafted the manuscript. HT carried out the *in situ *hybridization analysis. NL and YW carried out the bioinformatics analysis. CL and XL participated in the real-time RT-PCR assays. FS conceived of the study, and participated in its design and coordination and helped to draft the manuscript. All authors read and approved the final manuscript.

## Supplementary Material

Additional file 1 Differentially up-regulated miRNAs in NOA patients. The data provided represent the miRNAs that were found to be up-regulated in NOA patients compared to controls. Click here for file

Additional file 2 Differentially down-regulated miRNAs in NOA patients. The data provided represent the miRNAs that were found to be down-regulated in NOA patients compared to controls. Click here for file

Additional file 3 Several differentially expressed miRNA clusters in NOA patients. The data provided represent the miRNAs clusters that were found to be differentially expressed in NOA patients compared to controls. Click here for file

## References

[B1] Okada H, Tajima A, Shichiri K, Tanaka A, Tanaka K, Inoue I (2008). Genome-Wide Expression of Azoospermia Testes Demonstrates a Specific Profile and Implicates ART3 in Genetic Susceptibility. PLoS Genet.

[B2] Sun F, Turek P, Greene C, Ko E, Rademaker A, Martin RH (2007). Abnormal progression through meiosis in men with nonobstructive azoospermia. Fertil Steril.

[B3] Sun F, Trpkov K, Rademaker A, Ko E, Martin RH (2005). Variation in meiotic recombination frequencies among human males. Hum Genet.

[B4] Sun F, Kozak G, Scott S, Trpkov K, Ko E, Mikhaail-Philips M, Bestor TH, Moens P, Martin RH (2004). Meiotic defects in a man with non-obstructive azoospermia: case report. Hum Reprod.

[B5] Costa Y, Speed RM, Gautier P, Semple CA, Maratou K, Turner JM, Cooke HJ (2006). Mouse MAELSTROM: the link between meiotic silencing of unsynapsed chromatin and microRNA pathway?. Hum Mol Genet.

[B6] Stark A, Bushati N, Jan CH, Kheradpour P, Hodges E, Brennecke J, Bartel DP, Cohen SM, Kellis M (2008). A single Hox locus in Drosophila produces functional microRNAs from opposite DNA strands. Genes Dev.

[B7] Ro S, Park C, Sanders KM, McCarrey JR, Yan W (2007). Cloning and expression profiling of testis-expressed microRNAs. Dev Biol.

[B8] Kotaja N, Bhattacharyya SN, Jaskiewicz L, Kimmins S, Parvinen M, Filipowicz W, Sassone-Corsi P (2006). The chromatoid body of male germ cells: similarity with processing bodies and presence of Dicer and microRNA pathway components. Proc Natl Acad Sci USA.

[B9] Kotaja N, Sassone-Corsi P (2007). The chromatoid body: a germ-cell-specific RNA-processing centre. Nat Rev Mol Cell Biol.

[B10] Yu Z, Raabe T, Hecht NB (2005). MicroRNA Mirn122a reduces expression of the posttranscriptionally regulated germ cell transition protein 2 (Tnp2) messenger RNA (mRNA) by mRNA cleavage. Biol Reprod.

[B11] Hayashi K, Chuva de Sousa Lopes SM, Kaneda M, Tang F, Hajkova P, Lao K, O'Carroll D, Das PP, Tarakhovsky A, Miska EA, Surani MA (2008). MicroRNA Biogenesis Is Required for Mouse Primordial Germ Cell Development and Spermatogenesis. PLoS ONE.

[B12] Griffiths-Jones S, Saini HK, van Dongen S, Enright AJ (2008). miRBase: tools for microRNA genomics. Nucleic Acids Res.

[B13] Griffiths-Jones S, Grocock RJ, van Dongen S, Bateman A, Enright AJ (2006). miRBase: microRNA sequences, targets and gene nomenclature. Nucleic Acids Res.

[B14] Karolchik D, Kuhn RM, Baertsch R, Barber GP, Clawson H, Diekhans M, Giardine B, Harte RA, Hinrichs AS, Hsu F, Kober KM, Miller W, Pedersen JS, Pohl A, Raney BJ, Rhead B, Rosenbloom KR, Smith KE, Stanke M, Thakkapallayil A, Trumbower H, Wang T, Zweig AS, Haussler D, Kent WJ (2008). The UCSC Genome Browser Database: 2008 update. Nucleic Acids Res.

[B15] Sanger Institute. http://microrna.sanger.ac.uk/targets/v5/.

[B16] TargetScanHuman. http://www.targetscan.org/.

[B17] DIANA-microTest. http://www.diana.pcbi.upenn.edu/cgi-bin/micro_t.cgi.

[B18] PicTar. http://pictar.mdc-berlin.de/.

[B19] MiRGen. http://www.diana.pcbi.upenn.edu/miRGen.html.

[B20] Obernosterer G, Martinez J, Alenius M (2007). Locked nucleic acid-based in situ detection of microRNAs in mouse tissue sections. Nat Protoc.

[B21] Dillon N (2004). Heterochromatin structure and function. Biol Cell.

[B22] Bentwich I, Avniel A, Karov Y, Aharonov R, Gilad S, Barad O, Barzilai A, Einat P, Einav U, Meiri E, Sharon E, Spector Y, Bentwich Z (2005). Identification of hundreds of conserved and nonconserved human microRNAs. Nat Genet.

[B23] Tanzer A, Stadler PF (2004). Molecular evolution of a microRNA cluster. J Mol Biol.

[B24] Rockett JC, Patrizio P, Schmid JE, Hecht NB, Dix DJ (2004). Gene expression patterns associated with infertility in humans and rodent models. Mutat Res.

[B25] Braun RE (1998). Post-transcriptional control of gene expression during spermatogenesis. Semin Cell Dev Biol.

[B26] Altuvia Y, Landgraf P, Lithwick G, Elefant N, Pfeffer S, Aravin A, Brownstein MJ, Tuschl T, Margalit H (2005). Clustering and conservation patterns of human microRNAs. Nucleic Acids Res.

[B27] Voorhoeve PM, le Sage C, Schrier M, Gillis AJ, Stoop H, Nagel R, Liu YP, van Duijse J, Drost J, Griekspoor A, Zlotorynski E, Yabuta N, De Vita G, Nojima H, Looijenga LH, Agami R (2006). A genetic screen implicates miRNA-372 and miRNA-373 as oncogenes in testicular germ cell tumors. Cell.

[B28] Novotny GW, Sonne SB, Nielsen JE, Jonstrup SP, Hansen MA, Skakkebaek NE, Rajpert-De Meyts E, Kjems J, Leffers H (2007). Translational repression of E2F1 mRNA in carcinoma in situ and normal testis correlates with expression of the miR-17-92 cluster. Cell Death Differ.

[B29] Lin WW, Lamb DJ, Lipshultz LI, Kim ED (1999). Demonstration of testicular apoptosis in human male infertility states using a DNA laddering technique. Int Urol Nephrol.

[B30] Zeng Y, Rosborough RC, Li Y, Gupta AR, Bennett J (1998). Temporal and spatial regulation of gene expression mediated by the promoter for the human tissue inhibitor of metalloproteinases-3 (TIMP-3)-encoding gene. Dev Dyn.

[B31] Schumacher V, Gueler B, Looijenga LH, Becker JU, Amann K, Engers R, Dotsch J, Stoop H, Schulz W, Royer-Pokora B (2008). Characteristics of testicular dysgenesis syndrome and decreased expression of SRY and SOX9 in Frasier syndrome. Mol Reprod Dev.

[B32] Ying J, Srivastava G, Hsieh WS, Gao Z, Murray P, Liao SK, Ambinder R, Tao Q (2005). The stress-responsive gene GADD45G is a functional tumor suppressor, with its response to environmental stresses frequently disrupted epigenetically in multiple tumors. Clin Cancer Res.

[B33] Martin RH (2006). Meiotic chromosome abnormalities in human spermatogenesis. Reprod Toxicol.

[B34] Lamb NE, Hassold TJ (2004). Nondisjunction – a view from ringside. N Engl J Med.

[B35] Sun F, Greene C, Turek PJ, Ko E, Rademaker A, Martin RH (2005). Immunofluorescent synaptonemal complex analysis in azoospermic men. Cytogenet Genome Res.

[B36] Marcon E, Babak T, Chua G, Hughes T, Moens PB (2008). miRNA and piRNA localization in the male mammalian meiotic nucleus. Chromosome Res.

